# Ischemic Retinopathy and Neovascular Proliferation Secondary to Severe Head Injury

**DOI:** 10.1155/2014/410289

**Published:** 2014-07-20

**Authors:** Muge Coban-Karatas, Rana Altan-Yaycioglu

**Affiliations:** Department of Ophthalmology, Faculty of Medicine, Baskent University Adana, Dadaloglu Mahallesi Serinevler 2591 Sokak No. 4/A, Yuregir, 01250 Adana, Turkey

## Abstract

We report a case with severe head trauma and perforating globe injury in one eye and ischemic retinopathy and neovascular proliferation in the other eye. A 37-year-old male was brought to the emergency department after a motor vehicle accident with severe maxillofacial trauma. Ophthalmic examination revealed hematoma of the left eyelids as well as traumatic rupture and disorganization of the left globe. On the right eye, anterior segment and fundoscopic examination were normal. Primary globe repair was performed. At postoperative one-month visit, the right eye revealed no pathology of the optic disc and macula but severe neovascularization in the temporal peripheral retina. The patient was diagnosed as ischemic retinopathy and neovascular proliferation due to head trauma.

## 1. Introduction

Perforating globe injuries can cause severe ocular trauma with poor visual and anatomic outcomes [1]. Motor vehicle accidents are the most frequent cause of perforating globe injuries in young adults [2]. There are also several pathologies like Purtscher's retinopathy, Terson's syndrome, and sympathetic ophthalmia that should be taken into consideration in evaluation of the other eye that is not associated with trauma [3–5].

Herein, we report a case of perforating injury in one eye and peripheral retinal ischemia associated with peripheral retinal neovascularization in the fellow eye caused after a motor vehicle accident.

## 2. Case Report

**Figure 1:**
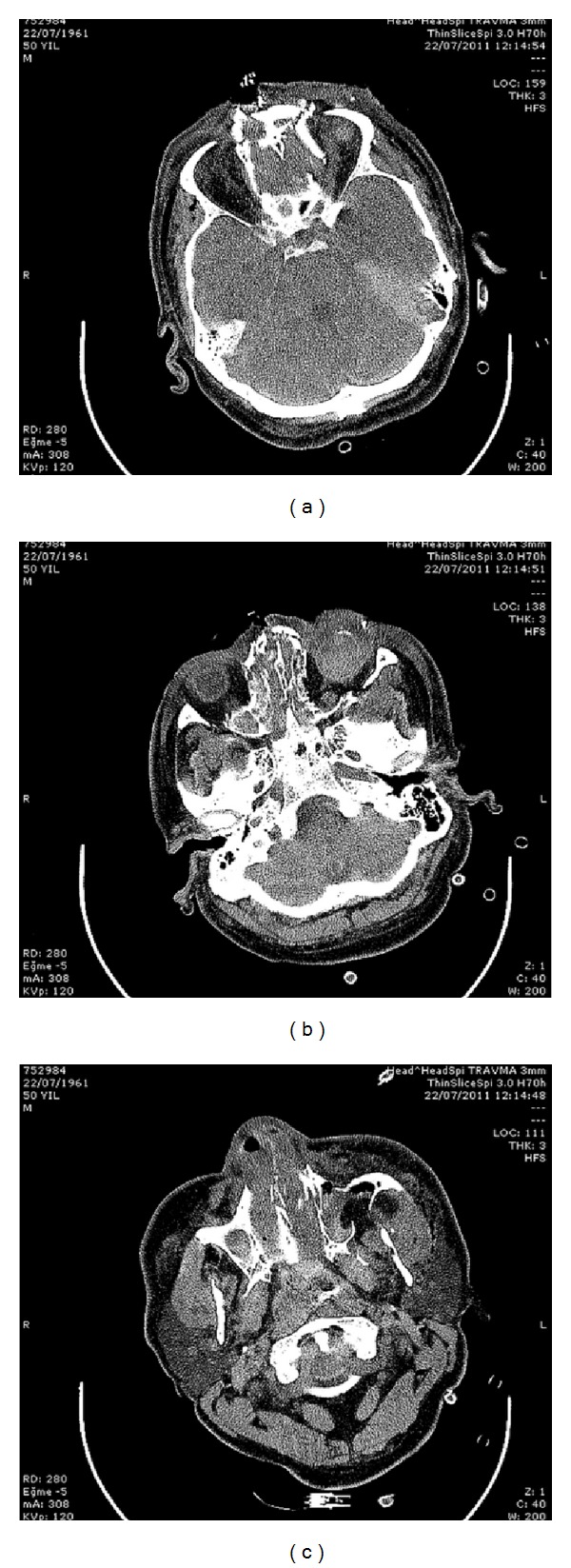
Head CT showing multiple fractures of the face. The patient suffered from depression fracture in the frontal (a), orbital and nasal (b), and maxillary (c) bones.

**Figure 2:**
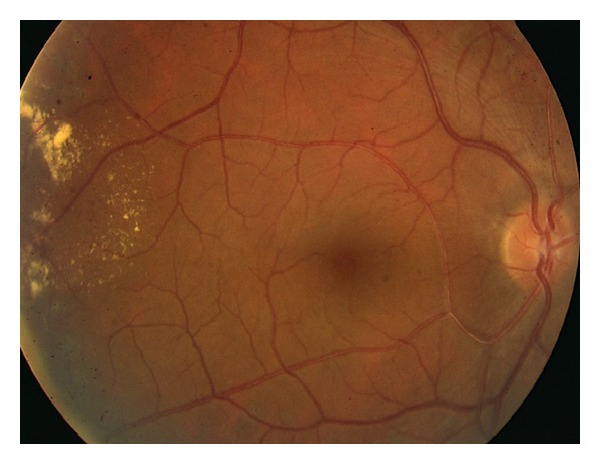
Fundus examination revealed no pathology of the optic disc and macula but severe neovascularization in the temporal peripheral retina.

**Figure 3:**
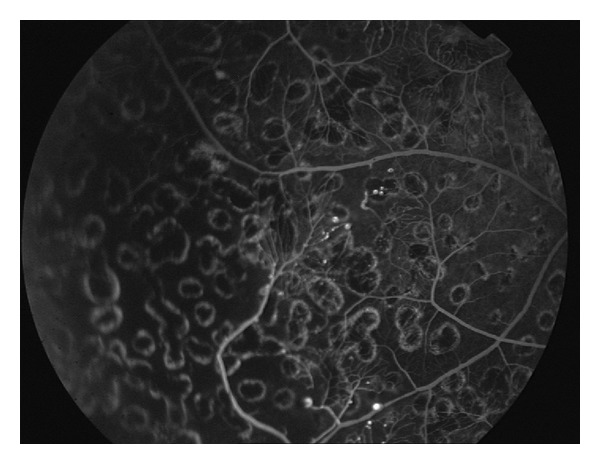
Argon laser photocoagulation was performed to the ischemic areas of the temporal peripheric retina of the right eye.

A 37-year-old male was brought to the emergency department after a motor vehicle accident with severe maxillofacial trauma. In the primary assessment, computerized tomography revealed head trauma with subarachnoidal bleeding, left frontal contusio cerebri, left parietooccipital subdural hematoma, and frontal and maxillofacial depression fracture (Figures 1(a), 1(b), and 1(c)). He was admitted to intensive care unit immediately. Ophthalmic examination revealed severe hematoma of the eyelids as well as traumatic rupture and disorganization of the left globe. Anterior segment and fundoscopic examination of the right eye were normal. Primary globe repair was performed immediately. He remained in the intensive care unit and received medical treatment. During his hospitalization in the intensive care unit no systemic diseases like diabetes, arterial hypertension, and blood cell and plasma disorders were detected. Cardiothoracic examination revealed no trauma. After one week his general condition improved and he was discharged three weeks following trauma. He had phthisis and hypoglobus in the left eye and enucleation and dermal fat graft implantation were performed to his no light perception left eye. In the right eye, his best corrected visual acuity was 1.0, and intraocular pressure was 14 mmHg. On slit lamp anterior segment and vitreous were normal. Fundus examination revealed no pathology of the optic disc and macula but neovascularization in the temporal peripheral retina (Figure 2). Fundus fluorescein angiography showed ischemia and hyperfluorescence due to capillary dysfunction in the peripheral retina. The patient was diagnosed as ischemic retinopathy and neovascular proliferation due to head trauma. Argon laser photocoagulation was performed to the ischemic areas (Figure 3). His right eye stayed stable in the follow-up of 28 months. Six weeks after dermal fat graft implantation, following confirmation of socket healing, prosthesis was inserted in his left eye.

## 3. Discussion

Ischemia of the retina is rarely reported following trauma of the head. In cases with perforating injuries to one eye sympathetic ophthalmia may be expected in the fellow eye. Our patient suffered from severe head trauma and perforating globe injury in one eye with phthisis and hypoglobus. The fellow eye revealed no pathology, but severe peripheral neovascularization due to capillary dysfunction.

Ischemic retinopathy and neovascular proliferation were described before secondary to shaken baby syndrome and nonaccidental trauma [6, 7]. Caputo et al. [6] reported three cases of peripheral retinal nonperfusion and ischemia associated with preretinal neovascularization after shaken baby syndrome. Although it is widely known that brain lesions occurring after baby shaking result from violent rotational acceleration-deceleration movements (whiplash movements) [8], some investigators postulate that ischemia could explain most of these lesions [9].

Dalma-Weiszhausz et al. reported 5 cases of retinal vascular occlusions following ocular contusion, found in FFA in otherwise healthy individuals. These patients suffered from retinal vascular occlusions following ocular contusion. All cases showed occlusion of the terminal vessels. The authors mention that the pathophysiology of these occlusions may involve the disruption of the endothelium from acute stretching of the retinal vessels due to the sudden deformation of the eye [10].

In differential diagnosis we evaluated Purtscher's retinopathy which is an occlusive microvasculopathy generally occurring as a result of cranial trauma or thoracic compression. The diagnosis is clinical, with sudden vision loss of variable severity. Fundoscopic signs include cotton-wool spots and intraretinal hemorrhages [3]. Terson's syndrome is the presence of intraocular hemorrhage in the setting of acutely elevated intracranial pressure due to intracranial injury [4]. Our patient also suffered from severe head trauma which may cause Purtscher's retinopathy and Terson's syndrome. In his fundoscopic examination cotton-wool spots and intraretinal or intraocular hemorrhages were not observed.

Our case suffered from subarachnoidal bleeding, contusio cerebri, subdural hematoma, and depression fracture in addition to perforating globe trauma in his left eye. His fundoscopic examination revealed no pathology in the fellow eye at optic disc and macula. On the contrary peripheral retinal ischemia, with neovascularization, was detected in the fellow eye. In cases with severe head and ophthalmic trauma, we would like to emphasize the importance of the continuing examination of the peripheral retina of the fellow eye.
